# PREDICTORS OF NURSING HOME COVID-19 CASES: A COMMUNITY VULNERABILITY APPROACH

**DOI:** 10.1093/geroni/igac059.032

**Published:** 2022-12-20

**Authors:** Matthew Peterson, Larry Lawhorne

**Affiliations:** UNCW College of Health and Human Services, Wilmington, North Carolina, United States; Wright State University, Fairborn, Ohio, United States

## Abstract

This study examined facility and community factors that were related to incident COVID-19 cases in nursing facilities. N=12,473 US nursing facilities were included in this study. Data from June 2020 - January 2021 from several sources were combined to create a dataset that included facility and community factors. Results indicated that higher staff shortages, poorer facility rating, for-profit ownership, proportionally more Medicaid and non-white residents were all significantly associated with higher COVID case rates over 8 months (all P < 0.0001). Community level predictors of higher cases included urban setting and higher Social Vulnerability Index (SVI). SVI was the strongest predictor of COVID case counts. This study assists in determining critical facility and community factors that predict increasing COVID burden in nursing facilities. Particularly, SVI is an important factor in determining facility and public health policy, and for targeting resources in large scale health crises such as the COVID-19 pandemic.

